# Risk of Severe Infections Secondary to the Use of Targeted Therapies in Hematological Malignancies

**DOI:** 10.7759/cureus.52050

**Published:** 2024-01-10

**Authors:** Mihaela Andreescu

**Affiliations:** 1 Department of Clinical Sciences, Titu Maiorescu University of Bucharest, Bucharest, ROU; 2 Hematology, Colentina Clinical Hospital, Bucharest, ROU

**Keywords:** chemotherapy., prophylaxis, mucosal barrier injury, immunosuppression, secondary immunodeficiency

## Abstract

Concurrent infections in hematological malignancies (HM) are major contributors to adverse clinical outcomes, including prolonged hospitalization and reduced life expectancy. Individuals diagnosed with HM are particularly susceptible to infectious pathogens due to immunosuppression, which can either be inherent to the hematological disorder or induced by specific therapeutic therapies. Over the years, the treatment paradigm for HM has witnessed a tremendous shift from broad-spectrum treatment approaches to more specific, targeted therapies. Even now, the therapeutic landscape of HM is constantly evolving due to the advent of novel targeted therapies and enhanced utilization of these agents for treatment purposes. By initiating unique molecular pathways, these agents hinder the proliferation of malignant cells, consequently affecting innate and adaptive immunity, which increases the risk of infectious complications. Due to the complexity of novel targeted therapies and their associated risk of infection, it often becomes a daunting task for physicians to maintain updated knowledge in their clinical practice. The situation is further aggravated by the fact that most of the initial clinical trials on targeted therapies provide inadequate information to conclude the associated risk of infection. In such a scenario, a cumulative body of evidence is paramount for guiding clinicians regarding the infectious complications that can arise following targeted therapies. In this review, I summarize the recent knowledge on infectious complications arising in targeted therapies for HM.

## Introduction and background

Infections remain a substantiated concern in patients with targeted therapies for hematological malignancies (HM) [1]. Patients with HM are inherently susceptible to infectious pathogens due to their impaired immune response, either as a direct result of their underlying hematological condition or as a consequence of specific therapeutic interventions aimed at targeting the malignancy [2]. Over the years, the treatment paradigm for hematological malignancies has witnessed a tremendous shift from broad-spectrum treatment approaches to more specific, targeted therapies that modify one or more cellular pathways [3]. Even now, targeted therapies remain at the forefront of ongoing research in hematological malignancies and are constantly reshaping the therapeutic landscape with novel therapeutic agents [4,5]. Initially, it was believed that the inception of these novel agents would minimize the infectious complication post-therapy. However, several unpredictable infectious sequelae have emerged with the use of some of the targeted therapies. Although targeted therapeutic agents demonstrate a narrow spectrum of toxicity primarily due to their specific signaling pathways, they have the potential to cause downstream path inhibition which can alter the immune system [6]. Consequently, prolonged immunosuppression in such patients exposes them to opportunistic pathogens. A broad array of pathogenic agents such as fungi, protozoa, and viruses have been identified in HM patients undergoing some targeted therapies [7,8].

Due to the constantly changing therapeutic landscape of HM and the advent of novel targeted therapies, it has become a daunting task for clinicians to keep track of potential infectious complications that can arise after treatment. Generally, it has been noted that most infectious disease physicians often exhibit a lack of comprehensive understanding regarding the fundamental physiological processes or untoward effects associated with the use of specific targeted therapies [9]. Therefore, it is critical for them to develop a deeper understanding and maintain updated knowledge regarding the unique risks that are associated with targeted therapies. However, most clinical studies reporting infectious complications in hematological malignancies provide incomplete data that rarely provide a structured presentation [10]. In such a scenario, a cumulative body of evidence is paramount for guiding clinicians regarding the infectious complications that can arise following targeted therapies. In this review, I summarize recent innovations, from an infectious complication perspective, regarding targeted therapies in hematological malignancies. I will cover targeted therapies that are frequently utilized in HM treatment including different monoclonal antibodies, bispecific T-cell engagers (BiTE), Bruton’s tyrosine kinase (BTK) inhibitors, Janus-associated kinase (JAK) inhibitors, and the B-cell lymphoma 2 (BCL-2) inhibitor (Table 1).

**Table 1:** List of targeted therapies in hematological malignancies and their risk of infection

Therapeutic intervention	Drugs	Mode of action	Approved indication	Risk of infection
CD20-targeted therapy	Rituximab obinutuzumab ofatumumab	Complement-dependent cytotoxicity, antibody-dependent cell-mediated cytotoxicity .	Non-Hodgkin's lymphoma (NHL) and chronic lymphocytic leukemia (CLL).	Rituximab treatment can result in severe infections including upper respiratory tract infections, sinusitis, nasopharyngitis, urinary tract infections, and bronchitis [11,12]. Viral infection with hepatitis B, cytomegalovirus infection, and the varicella-zoster virus has been observed in rituximab-treated patients [13].
CD38-targeted therapy	Daratumumab	Complement-dependent cytotoxicity, antibody-dependent cell-mediated cytotoxicity	Multiple myeloma	Neutropenia, thrombocytopenia, and anemia had been recorded with the use of daratumumab [14]. Most infections with daratumumab are of mild severity (grade 1 or 2) [15]. Patients undertaking daratumumab therapy are prone to varicella-zoster virus (VZV) infection [16].
CD52-targeted therapy	Alemtuzumab	Complement-dependent cytolysis (CDC) and antibody-dependent cellular cytotoxicity	Chronic lymphatic leukemia	Alemtuzumab leads to grade 3/4 neutropenia, thrombocytopenia, non-cytomegalovirus, cytomegalovirus infections, and anemia in chronic lymphocytic leukemia (CLL) patients [17].
CD19-targeted therapy	Inebilizumab	Modulates B cell receptor (BCR)-dependent and independent signaling pathways.	Acute lymphocytic leukemia	Inebilizumab-related infections included nasopharyngitis, upper respiratory tract infection, urinary tract infections, and hypertension [18].
Bispecific T-Cell Engagers (BiTE)	Blinatumomab	It crosslinks CD3 on T cells with CD19 antigen on B cells, consequently resulting in the activation of T cells and proliferation of cytolytic proteins to eliminate CD19-positive B cells.	Refractory acute lymphoid leukemia	The likelihood of serum IgG levels to normal is very bleak after blinatumomab treatment [19].
Kinase inhibitors	Ibrutinib, acalabrutinib, zanubrutinib	Inhibit Bruton tyrosine kinase (BTK)	Mantle cell lymphoma, chronic lymphocytic leukemia, Waldenström macroglobulinemia.	Acalabrutinib demonstrates a better safety profile which is associated with a significantly lower relative risk of infections in acalabrutinib-treated patients compared to non-acalabrutinib-based therapies [16]. A similar safety profile has been depicted by zanubrutinib [20]. Ibrutinib use is correlated with various infections such as diarrhea, upper respiratory tract infection, pyrexia, pneumonia, musculoskeletal pain, and atrial fibrillation. Hematological adverse events (AE)s include thrombocytopenia neutropenia, and anemia [21].
Phosphoinositide 3-Kinase (PI3K) Inhibitors	Idelalisib, duvelisib and copanlisib	Inhibition of PI3K pathways	chronic lymphocytic leukemia (CLL) and follicular lymphoma (FL).	Following idelalisisb, almost 32.1% (36/112) of patients experienced one or more infections. Viral infections/ reactivations were observed in 61·5% (16/26) of patients with a major share of cytomegalovirus (CMV) infection [22].
Janus-Associated Kinase (JAK) Inhibitors	Ruxolitinib	Inhibitor of Janus-associated kinases (JAKs).	Polycythemia vera	Ruxolitinib treatment was associated with grade 3/4 anemia and thrombocytopenia in 2% and 5% of participants, respectively whereas corresponding percentages were 0% and 4% in standard therapy [23].
BCL-2	Venetoclax	Selective inhibitors of the anti-apoptotic protein B-cell lymphoma 2 (Bcl-2)	Chronic lymphocytic leukemia, acute myeloid leukemia	Venetoclax use has been attributed to an increased risk of infections, mainly due to neutropenia [24]. Severe adverse events including sepsis, bacteremia, lung infection, and respiratory problems have been observed within 30 days of the first venetoclax [25].

## Review

Monoclonal antibodies

Due to their higher specificity and low adverse reactions, therapeutic monoclonal antibodies have emerged as the predominant drugs in the development phase [26]. As of 2022, eighty monoclonal antibodies have been approved by the Food and Drug Administration (FDA) for use [27,28]. In order to facilitate the distinction between various monoclonal antibodies, an international nomenclature has been proposed by using specific suffix corresponding to their biological origin such as murine, chimeric, humanized, or human antibodies [29]. Murine antibodies (suffix “-omab”) was the first to be formed from rodent sequences. However, since then utilization of more sophisticated engineering technologies has led to more specified antibodies such as chimeric (suffix “-ximab”) from the combination of murine Fab and human Fc regions [30]. Humanized antibodies are denoted by the suffix “-zumab” and are primarily formed by human sequences but have complementarity-determining regions (CDRs) of murine origin. Human antibodies (suffix “-umab”) demonstrate lower immunogenic properties and are developed purely from human sequences [31].

Anti-CD20 monoclonal antibodies

The inception of anti-CD20 monoclonal antibodies (mAbs) was hailed as a groundbreaking event [32,33]. Anti-CD20 mAbs depict a family of treatment therapies targeted at CD20-positive B-cell malignancies and other orphan autoimmune diseases [34,35]. As CD20 is a B cell-specific membrane protein that is expressed on normal and malignant B cells but not on B-cell precursor or plasma cells, anti-CD20 mAbs do not cause immediate impairment in immunoglobulin production [36]. Repeated doses of some anti-CD20 antibodies have been associated with hypogammaglobulinemia and the late onset of neutropenia (LON) [37]. The underlying mechanism of immunosuppression by anti-CD20 mAbs includes long-lasting B-cell depletion, either by apoptosis or cell-mediated cytotoxicity, which consequently results in alterations in humoral immune response [38]. Following anti-CD20 monoclonal antibody treatment, a decrease in the B cell population may persist for at least 6 to 9 months [9]. Further anti-CD20 antibodies impart various downstream effects by influencing the function of B- and T-cells regarding antigen presentation and cytokine production [39].

Rituximab

Rituximab was the first anti-CD20 monoclonal antibody that was approved for the treatment of lymphoid malignancy [40]. It is of chimeric origin and binds to the CD20 antigen present in all peripheral B cells. Rituximab is indicated for relapsed/refractory, follicular B-cell Non-Hodgkin’s Lymphoma (NHL), newly diagnosed or previously treated CLL, microscopic polyangiitis (MP), rheumatoid arthritis, and systemic lupus erythematosus (SLE) [41-46]. Late onset of neutropenia has emerged as a frequently reported consequence of rituximab either as monotherapy or combination therapy [47]. Shimony et al. shared the findings from 330 study participants with lymphoproliferative neoplasms who were categorized into rituximab (n = 283) and obinutuzumab (n = 47) treatment groups [48]. Late onset of neutropenia was observed in 23% of patients who were present in the rituximab arm of the study [48]. Similar results were shared by Tesfa et al. who investigated 169 evaluable consecutive rituximab-treated NHL patients. Fifteen patients (9%) in the treatment group developed late-onset neutropenia (LON). They also evaluated the levels of different cytokines (G-CSF, SDF1, BAFF, APRIL) to understand the underlying mechanism of rituximab-induced LON. They observed transient bursts of blood granulocyte colony-stimulating factor (G-CSF) and serum B cell activating factor from the tumor necrosis factor family (BAFF) concentrations in LON patients which can partially explain the rituximab-induced LON as neutrophils are a major source of BAFF and their release is initiated by G-CSF [49-51]. However, the complete mechanism of LON following rituximab therapy remains poorly understood. In the majority of cases, neutropenia induced by rituximab therapy resolves spontaneously [52].

Hypogammaglobulinemia is another major concern that has been reported after rituximab therapy [53]. Low levels of immunoglobulins are a significant contributor to infectious complications as they exhibit a prominent role in protective immunity. Tiu et al. evaluated long-term clinical outcomes following rituximab therapy in 142 patients who had autoimmune diseases [54]. Their findings showed a median time of 22.5 months with IgG <5g/L in rituximab-treated patients [54]. Casulo et al. observed an association between rituximab administration and an increased risk of symptomatic hypogammaglobulinemia [55]. Almost 39% of their study participants who received multiple rituximab courses had low levels of IgG whereas 6.6% developed recurrent sinopulmonary infections. However, their conditions improved after intravenous immunoglobulin therapy [55]. A systemic review by Arnold et al. showed that rituximab resulted in serious infections in 7 (2.3%) of the total 303 patients, of which 4 had fatal outcomes [56]. Cohen et al., in their phase 3 trial, evaluated the safety and efficacy of rituximab at twenty-four weeks of assessment. They reported that the rate of serious infection was 5.2/100 patient-year following rituximab treatment compared to 3.7 in the placebo group. The most common infections in the rituximab group included upper respiratory tract infections, sinusitis, nasopharyngitis, urinary tract infections, and bronchitis [11].

A review of the literature by Aksoy et al. investigated 64 cases of rituximab-related viral infections in lymphoma patients. They found that hepatitis B was the most frequent viral infection, followed by cytomegalovirus infection, and varicella-zoster virus [12]. Rituximab has also demonstrated higher infection rates in combination therapy either with chemotherapy or immunotherapy. A CLL-10 trial by Eichhorst et al. evaluated two treatment approaches for advanced chronic lymphocytic leukemia (CLL) with fludarabine (F), cyclophosphamide (C), and rituximab (R) (FCR) compared to bendamustine (B) and rituximab (BR). His findings demonstrated that FCR was superior in terms of efficacy compared to BR; however, FCR was associated with significantly higher severe infections compared to the BR group. The risk of infection was higher in participants aged over 65 years and infections occurred late during treatment which can be explained by LON [57]. A case report of 31-year-old women, who received combination therapy with cyclophosphamide and rituximab for indolent lymphoma, showed depressed CD4 levels and panhypogammaglobulinema while having recurrent sinus infections [58]. However, the symptoms improved after monthly intravenous immunoglobulin treatments [58].

A phase 3 study evaluated the 6-year outcome in rituximab maintenance treatment for resistant follicular NHL. Their findings showed that survival was improved to 74% in the rituximab treatment arm compared to 64% in the observation arm. However, rituximab maintenance for NHL was significantly associated with grades 3/4 infections (9.7% v 2.4%). At the 2-year evaluation, in the observation arm, serum immunoglobulin (Ig) G levels increased from 6.6g/L to 7.3g/L whereas, in the treatment arm, it was 6.5 g/L at the 2-year assessment, and 6.3 g/L at the end of maintenance therapy [59]. Moulis et al. performed a large population study including patients with immune thrombocytopenia to evaluate the risk of infections after rituximab treatment. Their findings showed that the serious infection rate for the lower respiratory tract was 42.8%, whereas the treatment group had almost 2.6 times more risk of developing serious bacterial and viral infections compared to the placebo [60]. A high prevalence of hepatitis C virus (HCV) infection has been described in B-cell non-Hodgkin's lymphoma patients. Marignani et al., in a retrospective analysis of 104 consecutive patients, found that 9 (8.6%) were HCV positive, with no reported death at 12-month follow-up [61].

Infections with opportunistic pathogens such as *Pneumocystis jiroveci* post-rituximab treatment therapy have been reported in the literature due to impaired cell-mediated immunity. A systemic review of 11 cohort studies showed that lymphoma treated with a rituximab regimen was significantly associated with the risk of pneumocystis pneumonia (PCP) (risk ratio: 3.65) [62]. However, the incidence of PCP was reported to be very low by Barreto et al. in patients with B cell lymphoma who were treated with rituximab. They analyzed a total of 689 patients after 180 days of the last treatment therapy and found a PCP incidence of 1.51% which was even below the conventional threshold for considering the use of prophylaxis [63]. According to the guidelines of the 5th European Conference on Infections in Leukemia (ECIL-5), trimethoprim/sulfamethoxazole should be given 2-3 times every week for prophylaxis of PCP during at-risk periods after rituximab therapy [64]. The management of CLL has been targeted with anti-CD20 mAbs [65]. Goede et al. compared the efficacy of obinutuzumab and rituximab in combination with chlorambucil in CLL patients. Their findings showed that rituximab addition was associated with grade 3/4 neutropenia (34%) and thrombocytopenia (11%) [66].

Ofatumumab

Ofatumumab is a fully humanized anti-CD20 monoclonal antibody [67]. After binding to CD20, the Fc portion of ofatumumab induces cytolysis of B cells [68]. A phase 3 trial by Byrd et al. evaluated the safety and efficacy of ofatumumab compared to ibrutinib in 391 patients with refractory CLL. Although the incidence of grade 3 or 4 infections was similar in both groups, ofatumumab showed a lower number of infections compared to ibrutinib (54% vs 70%). Common adverse reactions included rash (8% vs. 4%), pyrexia (24% vs. 15%), and blurred vision (10% vs. 3%) in ibrutinib and ofatumumab, respectively [13]. A phase 3 trial by Davids et al. compared the safety and efficacy of ofatumumab and duvelisib in patients with relapsed/refractory (R/R) CLL/small lymphocytic lymphoma (SLL) [69]. Their findings showed that adverse events of grade 3/4 were more common in ofatumumab compared to duvelisib including diarrhea (47%/23%), pyrexia (24%/4%), cutaneous reactions (23%/4%), and thrombocytopenia (10%/6%); however, neutropenia was similar in both treatment groups (26%/23%) [69]. Desikan et al. reported that early treatment with ofatumumab in high-risk CLL patients is well-tolerated with only adverse events being related to infusion reactions which were amendable by antihistamine and/or steroid treatment [70].

Obinutuzumab

Obinutuzumab is a humanized monoclonal antibody that was approved in 2017 for the treatment of untreated CLL and untreated or R/R follicular lymphoma (FL) [71,72]. Obinutuzumab leads to cytolysis of B cells by activating complement and apoptotic pathways [73]. Marcus et al., in their randomized trial, categorized 1202 patients equally in each group of obinutuzumab-based chemotherapy and rituximab-based chemotherapy for follicular lymphoma [74]. At 34.5 months follow-up, a higher rate of infection (20%) was reported in the obinutuzumab-treated group compared to 15.6% in the rituximab group [74]. In a randomized control trial, Goede et al. compared the efficacy of obinutuzumab and rituximab, each combined with chlorambucil in CLL patients. The overall rate of grade 3/4 infections ranged between 11 to 14% and was indifferent between both groups; however, infusion-related adverse events and neutropenia were more prevalent in the obinutuzumab-treated arm of the study [75].

CD38-directed agents and risk of infection

The CD38 antigen represents a frequently expressed antigen on plasma cells which makes them an excellent target for treatment in multiple myeloma (MM) by anti-CD38 directed agents [76]. Daratumumab, an anti-CD38 antibody, has demonstrated efficacy in MM by inducing Fc-mediated cell lysis by cell-mediated toxicity and complement activation [77]. Consequently, daratumumab leads to the depletion of CD38-positive myeloid-derived suppressor cells, T-, and B-cells. Several adverse events including neutropenia, thrombocytopenia, and anemia had been recorded with the use of daratumumab [14]. Dimopoulos et al. recruited 569 patients with multiple myeloma to investigate the effects of daratumumab and a combination of lenalidomide with dexamethasone. Their findings showed that the severity of daratumumab-treated infections was mild (mostly grade 1 or 2) [14]. Similar findings were shared by Palumbo et al. in their phase 3 trial, who found that most infections were of grade 1 or 2 severity, with only 8.6% exhibiting grade 3 level infection [15]. Patients receiving CD38-targeted agents such as daratumumab can be more prone to varicella-zoster virus (VZV) infection [16]. A large phase 3 study was performed by Spencer et al. involving 498 patients with R/R MM [78]. In their CASTOR trial, participants were randomized to bortezomib and prednisone or daratumumab, bortezomib, and prednisone. Their findings demonstrated that the daratumumab arm of the study resulted in significantly prolonged neutropenia (12.8% versus 4.2%) and risk of infectious complications (21.4% versus 19.0%) [78]. Similarly, Bahlis et al. randomized 569 patients who were previously treated for multiple myeloma into daratumumab, dexamethasone, and lenalidomide or dexamethasone and lenalidomide alone [79]. They reported that the daratumumab group had higher neutropenia compared to the daratumumab-negative group (5.7% versus 2.5%) and serious pneumonia (8.1% versus 8.5%) [79].

CD52-directed agents

Alemtuzumab, a humanized monoclonal anti-CD52 antibody that binds to cell surface CD52 glycopeptide expressed almost on all human lymphocytes, monocytes, and macrophages, leads to the depletion of CD52-positive B and T cells [80]. Alemtuzumab induces antibody-dependent cell-mediated cytolysis which leads to the depletion of lymphocytes. Low circulating CD4+ lymphocyte counts persist for 1 to 2 years after alemtuzumab administration [81,82]. Alemtuzumab is often contraindicated in patients who are infected with human immunodeficiency virus (HIV), primarily due to depleted levels of CD4+ lymphocytes [83]. The immunosuppression caused by alemtuzumab can lead to the reactivation of hepatitis B virus (HBV) infection [84]. Findings of two cases of chronic lymphocytic leukemia with occult HBV infection by Lannitto et al. reported activation of HBV after immunotherapy with alemtuzumab [85]. Alemtuzumab has also been implicated in higher herpes infections. Cohen et al., in their phase 3 trial, assessed the comparative effects of alemtuzumab and Interferon Beta 1a [86]. Patients who were treated with alemtuzumab had higher rates of herpes infections compared to patients treated with Interferon Beta 1a (16% vs 2%) [86].

A phase 2 trial by Stilgenbauer et al. reported that alemtuzumab resulted in grade 3/4 neutropenia (56%), thrombocytopenia (57%), and anemia (49%) in CLL patients. Grades 3 to 4 non-cytomegalovirus and cytomegalovirus infections occurred in 29% and 8% of patients, respectively [17]. A smaller study by Poh et al., with only 5 participants receiving alemtuzumab, reported no cases of CMV infections [87]. CMV reactivation is attributed to the depletion of T cells following alemtuzumab treatment therapy [88]. A randomized trial by O'Brien et al. evaluated the efficacy of valganciclovir against reactivation of CMV post-alemtuzumab therapy. They showed that none of the patients from the treatment arm showed reactivation compared to 35% in the without prophylaxis group [89]. Although rare, mycobacterium tuberculosis has been reported in literature following alemtuzumab therapy. Kim et al. investigated the efficacy of alemtuzumab alone or alemtuzumab-containing chemotherapy. Their finding revealed that out of 182 study participants, 16 were positive for tuberculosis [90]. Other reported infectious complications in their trial included CMV (36%), varicella zoster virus (13%), and fungal infection (17%) [90]. Bosch et al. reported two cases of patients who received alemtuzumab as part of their renal transplant management and later developed *Mycobacterium tuberculosis *infection [91]. A pooled analysis of 6-year data from CAMMS223, CARE-MS I, and CARE-MS II studies, and the CAMMS03409 extension study revealed that the risk of infection with alemtuzumab is mostly mild or moderate with only 1.0%-1.9% serious infections per year. The findings showed that infections decrease over time due to the preservation of protective immunity with time [92].

CD19 targeted agents

Anti-CD19 monoclonal antibodies have demonstrated efficacy against several R/R B-cell malignancies [93-96]. The expression of CD19 is mostly restricted to the B cell population and it commences in early developmental stages. Almost all plasma B cells in peripheral circulation and around 50% of plasma cells in bone marrow express CD19 on their surfaces. Compared to CD20, it is expressed at an earlier stage [97]. Research has highlighted that infection can occur before and after B cell depleting therapies until there is complete recovery of serum immunoglobulins. Inebilizumab is a humanized anti-CD19 mAb that depletes lymphocytes derived from the B cell lineage [98]. Agius et al. evaluated the safety and tolerability of inebilizumab, an anti-CD19 monoclonal antibody agent. Their findings showed that inebilizumab caused a decrease in immunoglobulin levels and adverse reactions including nasopharyngitis (24%), upper respiratory tract infection (19%), urinary tract infection (14%), urinary tract inflammation (14%), pyrexia (14%) and increased blood pressure (14%). However, most infections were of grade 1 or 2 severity [18]. Recently, two novel anti-CD19 monoclonal antibodies have been approved by the FDA, including tafasitamab and loncastuximab tesirine, which have been propagated as viable options for the treatment of R/R diffuse large B-cell lymphoma (DLBCL). However, further studies are required to evaluate infectious complications with these therapies [99].

Bispecific T-Cell Engagers (BiTE)

Blinatumomab, a bispecific T cell engager (BiTE) antibody, was the first treatment for refractory acute lymphoid leukemia [100]. Blinatumomab crosslinks CD3 on T cells with CD19 antigen on B cells, consequently activating T cell production to eliminate CD19-positive B cells. As they are cytotoxic to CD19-positive cells invariably to their cancerous nature, they are likely to cause secondary antibody deficiency [101]. Although the number of T cells returns to base within 7 to 14 days, lower B cell levels are likely to persist throughout treatment. Consequently, it leads to hypogammaglobinemia which continues for over a year. Zugmaier et al. reported that the likelihood of serum IgG levels returning to normal is very bleak after blinatumomab treatment [102]. In their phase 2 study, they demonstrated that upon follow-up for 255 to 1605 days (median, 457.5 days) among six patients treated with blinatumomab, 5 out of 6 patients did not recover their IgG levels. Only one subject was able to obtain normalcy after 2 years. As CD19 is expressed on plasma blasts, their targeted therapy induced more profound immune suppression. They demonstrated that grade 3 infections were reported by two out of six of the participants [102].

A phase 3 RCT by Kantarjian et al. reported that almost 6% of participants reported depleted IgG levels treated with blinatumomab compared to 0.9% in the chemotherapy arm of the study [103]. However, these results were not translated to neutropenia outcomes, as blinatumomab caused lower neutropenia (38%) compared to 58% in the chemotherapy group, whereas AEs of grade 3 or more were also less frequent (87% in the blinatumomab group compared to 92% in chemotherapy arm of the study) [103]. Further characterization of blinatumomab-related infections was provided by Topp et al. in their phase II clinical trial [104]. They reported grade 3 infections in participants with a higher number of catheter-related infections (9.5%), followed by bacterial/Escherichia sepsis (4.8%), and bronchopneumonia (4.8%) [104]. Similar observations were shared by another phase 2 trial in patients aged over 65 years with relapsed/refractory B-precursor acute lymphoblastic leukemia (r/r ALL). They reported that grade 3 or more AEs were reported in 86% of participants, whereas infections were seen in 39% of patients [19].

Bruton’s Tyrosine Kinase (BTK) Inhibitors

The management of hematological disorders has undergone profound changes in recent years with the rise of novel anti-cancerous agents [105]. Several Bruton tyrosine kinase (BTK) inhibitors, including ibrutinib, acalabrutinib, and zanubrutinib, have emerged that inhibit Bruton tyrosine kinase (BTK). Ibrutinib, a first-in-class BTK drug, has been attributed to an increased population of activated T cells and diminished levels of Treg/CD4+ T cell ratio while imparting its immunomodulatory effects against CLL through inhibition of BTK and IL-2-inducible T cell kinase (ITK) [106]. However, ibrutinib use has shown several adverse reactions like diarrhea, upper respiratory tract infection, hyperuricemia, pyrexia, pneumonia, musculoskeletal pain, and atrial fibrillation. Severe infections of grade 3 or higher have been reported in 35% of the patients. The most commonly cited hematological AEs include thrombocytopenia, neutropenia, and anemia [21]. Acalabrutinib is a novel BTK inhibitor that is recommended for the treatment of CLL. The efficacy of acalabrutinib is well-established, with recent research demonstrating its better safety profile. A meta-analysis of 3 RCTs with 1362 patients reported a significantly lower relative risk of infections in acalabrutinib-treated patients compared to non-acalabrutinib-based therapies [107]. Another BTK inhibitor, zanubrutinib has demonstrated a better safety profile compared to other targeted agents. Investigations by Trotman et al. in 73 Waldenström macroglobulinemia patients concluded that long-term treatment with single-agent zanubrutinib demonstrated a durable response with an acceptable safety profile [20]. AEs mostly included grade 3 diarrhea, neutropenia, and atrial fibrillation [20]. A review by Tillman et al. reported that infectious complications such as pneumonia developed in 56% of patients taking single-agent ibrutinib and 52% of those on combination therapy [108].

Phosphoinositide 3-Kinase (PI3K) inhibitors

The activation of receptors on B cells leads to downstream signaling pathways that ensure proliferation, cell survival, and motility. Normally these pathways include phosphatidylinositol 3-kinase (PI3K), protein kinase B (AKT), and mammalian target of rapamycin (mTOR), and are often activated in B cell malignancies [109]. The activation of PI3K has been implicated in the recruitment of several intracellular enzymes that lead to cancerous cell proliferation [110]. Therefore, PI3K represents an important target for anticancer therapy in several hematological malignancies [111]. Idelalisib is an orally bioavailable, small molecule, reversible inhibitor of PI3K- δ [112]. It was the first PI3K inhibitor that was approved for the treatment of CLL and follicular lymphoma (FL) [113]. Other PI3K inhibitors, including duvelisib and copanlisib, were later approved. A phase 3 trial by Furman et al. evaluated idelalisib along with rituximab in the treatment of relapsed CLL. They reported that serious adverse events occurred in 40% of patients including pneumonia, pyrexia, and febrile neutropenia [114].

A phase 3 trial by Zelenetz et al. compared the addition of idelalisib or placebo to bendamustine and rituximab in patients with relapsed or refractory CLL. Their findings showed that 60% of the patients in the idelalisib arm developed neutropenia, whereas 23% demonstrated febrile neutropenia [115]. They also observed a higher frequency of infections in the idelalisib-treated group compared to the placebo group (69% versus 59%). Pneumonia of bacterial origin was reported in 14% of patients in the idelalisib arm of the study, and CMV infection (6%), PJP (2%), and pulmonary mycoses were also observed in the treated group [115]. Similar results were shared by Jones et al. who reported grade 3 or higher neutropenia and pneumonia in 34% and 14% of patients, respectively, with idelalisib treatment compared to 16% and 8% in ofatumumab monotherapy [116]. Lymphocytosis is often reported in patients who undergo PI3K inhibitors as monotherapy [117-119]. The SEIFEM retrospective study reported infectious complications with ibrutinib and idelalisib in lymphoproliferative disorders [22]. Almost 32.1% (36/112) of patients experienced one or more infections. Viral infections/ reactivations were observed in 61.5% (16/26) of patients with a major share having CMV infection [22]. PI3K inhibitors have demonstrated a variable risk of infection, with some depicting an acceptable risk of infection and others culminating in the termination of the trial owing to severe adverse reactions [120,121].

Janus-associated kinase (JAK) inhibitors

Janus-associated kinases (JAKs) are a family of four receptors that mediate the signaling of cytokine receptors via the signal transducer and activator of the transcription (STAT) pathway. They are involved in the proliferation of a variety of cells but play a crucial role in immune and hematopoietic cells [122]. Ruxolitinib, an inhibitor of JAK1 and JAK2, was approved in 2011 for the treatment of myelofibrosis. It leads to the downregulation of T-helper cell type 1 (Th1) responses and cytokines including IL-1, IL-6, and TNFα [123]. A phase 3 randomized trial by Vannucchi et al. investigated ruxolitinib versus standard therapy for polycythemia vera. Their findings showed that grade 3/4 anemia and thrombocytopenia occurred in 2% and 5% of participants, respectively, whereas corresponding percentages were 0% and 4% in standard therapy. Herpes zoster infection was much higher (6%) in the ruxolitinib-treated group compared to 0% in the standard therapy group [23]. Similar observations were shared by Verstovsek et al. in patients with myelofibrosis who underwent ruxolitinib treatment. They found that herpes zoster infections were more common in the ruxolitinib-treated group compared to the untreated group; however, other infectious complications were similar in both groups [124]. Similarly, a review and meta-analysis by Lussana et al. reported that ruxolitinib treatment was associated with a higher risk of herpes zoster infection compared to the control group [125].

B-Cell Lymphoma 2 (BCL-2) Inhibitors

BCL-2 inhibitors represent a class of anti-tumor agents that are selective inhibitors of the anti-apoptotic protein B-cell lymphoma 2 (Bcl-2). Upon binding to BCL-2, they inhibit its activity, which restores apoptotic processes in tumor cells [126,127]. Venetoclax has shown efficacy in the treatment of relapsed chronic lymphocytic leukemia. However, its use has been attributed to an increased risk of infections, mainly due to neutropenia. A clinical trial by Davids et al. observed a higher incidence of grade 3/4 neutropenia which resulted in infections in almost 15% of patients [24]. The safety analysis of 350 CLL patients showed that infection of any type was observed in 72% of patients with a major share of respiratory infections and fever [24]. These findings were supported by DiNardo et al. who reported severe adverse events including sepsis, bacteremia, lung infection, and respiratory problems within 30 days of the first venetoclax [25].

Other novel agents

As the therapeutic landscape of hematological malignancies is being enriched with novel targeted agents, there are several targeted agents are still in the process of safety evaluations. Due to a lack of substantial evidence, I have summarized them here. For example, brentuximab vedotin is a conjugated antibody directed against CD30 which was approved in 2011 for the treatment of Hodgkin’s lymphoma, R/R anaplastic lymphoma, and cutaneous T-cell lymphoma [128]. The major risk factor of infectious complications arises due to the tendency of this drug to cause neutropenia [129,130]. Tudesq et al. have reported cytomegalovirus infection after brentuximab vedotin treatment [131]. Inotuzumab ozogamicin is a CD22-directed antineoplastic agent that is used in the treatment of B-ALL [132,133]. Kantarjian et al. reported lower rates of neutropenia compared to standard therapy. However, veno-occlusive liver disease was observed in 11% (15/109) who received inotuzumab ozogamicin and in 1% in standard therapy [132]. FMS-like tyrosine kinase 3 (FLT3) inhibitors are novel agents that target FLT3, a receptor tyrosine kinase that is expressed primarily in the hematopoietic compartment [134]. Over 30-35% of patients suffering from acute myeloid leukemia are due to mutations of FLT3‐ITD and FLT3‐TKD, consequently resulting in prolonged activation of protein that promotes cell proliferation and survival [135]. A review by Xu et al. demonstrated that FLT3 inhibitors improved outcomes in the induction/reinduction stage of FLT3(+) AML; however, adverse reactions including thrombocytopenia, neutropenia, anemia, cardiac abnormalities, dyspnea, and cough [136]. The breakpoint cluster region/Abelson leukemia virus (BCR-ABL) inhibitors have been used to treat chronic myeloid leukemia (CML), acute lymphocytic leukemia (ALL), and other hematological malignancies. Imatinib was the first approved drug in this class for the treatment of CML or ALL [137]. Kalmanti et al. evaluated the 10-year safety and efficacy of imatinib in CML. Their findings showed that the eight-year probability of grade 3/4 adverse events was 22% [138]. Isocitrate dehydrogenase (IDH) inhibitors are another type of targeted therapies that target the genetic mutations in isocitrate dehydrogenase genes (IDH1 and IDH2) in acute myeloid leukemia (AML), occurring in up to 30% of AML cases [139]. Most infectious complications with these agents are mild in nature [140].

Impact of targeted therapies on SARS-CoV-2 infections

The intersection of coronavirus disease 2019 (COVID-19) infection and targeted therapies poses complex therapeutic dilemmas for healthcare providers as both cause significant morbidity and mortality. The interplay between the host immune system, underlying hematological malignancy, and targeted therapies can have significant impacts on the course of COVID-19 illness. Patients suffering from CLL have demonstrated an augmented vulnerability towards the severe manifestation of the novel coronavirus (COVID-19), irrespective of their disease phase or their current treatment regimen [141,142]. A joint retrospective international multicenter study by ERIC, the European Research Initiative on CLL, and the CLL Campus evaluated 190 patients with confirmed CLL and COVID-19 [143]. The majority of participants (79%) presented with severe COVID-19 (need of oxygen and/or intensive care admission). The rate of hospitalization was significantly lower in ibrutinib-treated patients (p-value < 0.05) compared to patients on alternate regimens [143]. However, these findings were not supported by Courtre et al., who found no improvement with the addition of ibrutinib in the routine standard of care [144].

Some studies have demonstrated impaired serologic response following COVID-19 vaccination in CLL patients undergoing targeted therapies, particularly anti-CD20 antibody therapy [145,146]. Herishanu et al. investigated the antibody response of the third dose of the BNT162b2 mRNA vaccine in CLL/SLL patients who failed to achieve a humoral response after standard second dose vaccination [147]. Antibodies against severe acute respiratory syndrome coronavirus 2 (SARS-CoV-2) were measured 3 weeks post-vaccination. Their findings revealed that 23.8% of the 172 CLL patients had an antibody response. The response rate was lower among patients who were actively treated (12.0%) compared to those who were treatment-naïve (40.0%) and off-therapy (40.6%). Furthermore, the lowest response rate was observed in patients receiving Bruton’s tyrosine kinase inhibitors or venetoclax with or without anti-CD20 antibody treatment (15.3% and 7.7%, respectively). Only a limited proportion of patients treated with anti-CD20 antibodies less than 12 months prior to vaccination (3.6%) demonstrated an antibody response [147]. Similarly, Parry et al. investigated spike-specific antibody responses following COVID-19 vaccination in 299 CLL patients and healthy subjects [145]. Their results showed that 34% of CLL patients demonstrated spike-specific antibody responses which were significantly lower compared to 94% of healthy participants with 104-fold lower antibody titers in the CLL group. After the second vaccine, the response rate increased to 75% in CLL patients but was lower when compared to the control group (100%) [146].

A study by Shen et al. assessed the immune response in 181 CLL and monoclonal B-cell lymphocytosis (MBL) patients in correlation with their seroconversion status following the administration of two doses of the SARS-CoV-2 spike protein IgG assay [148]. The results revealed that 79.2% of CLL patients and 50% of MBL patients failed to achieve seroconversion after the first dose, whereas 45% of CLL and 9.5% of MBL remained seronegative following the second dose. Univariate analysis indicated a significant correlation between the antibody level post-dose two and pre-vaccination levels of reduced IgM (p<0.0001), IgG2 (p<0.0351), and IgG3 (p<0.0457), as well as therapy received by CLL patients within the previous 12 months (p<0.001) [145]. Blixt et al. evaluated sixty consecutive CLL patients during the first 13 months of the pandemic. Seroconversion to anti-SARS-CoV-2 antibodies was observed in 82% of the 40 tested patients, with 17/22 and 8/11 patients testing positive for antibodies at 6 and 12 months, respectively [148]. The risk of COVID-19 in patients undergoing targeted therapies is an intriguing topic. However, we will not have a detailed discussion here as this is out of the scope of our review. As significant evidence has emerged in this regard, a separate review on this aspect will be better suited.

## Conclusions

The advent of novel targeted therapies has broadened the treatment horizons of hematological malignancies. This everchanging treatment landscape has brought several challenges to practicing clinicians that require updated knowledge to tackle a broad range of clinical manifestations arising following targeted therapies. Patients who receive targeted therapies for hematological malignancies are prone to infectious complications as some of these therapies have a profound impact on the immune status of the individuals. The risk of infection can vary among individuals owing to their underlying malignancy and previous therapeutic treatments. This highlights a need for a thorough compilation of knowledge on this subject, collating the clinical evidence of infectious complications in several targeted agents. However, several challenges emerge in this setting as most targeted therapies are used in combination with chemotherapy or other immunosuppressive agents such as glucocorticoids. This further exacerbates the challenge of identifying the attributable infection risk of one particular agent. The infectious consequences of targeted therapies can be better managed by adopting screening of latent infection and management practices for hematological malignancies.

## References

[1] Ruiz-Camps I, Aguilar-Company J (2021). Risk of infection associated with targeted therapies for solid organ and hematological malignancies. Ther Adv Infect Dis.

[2] Goldman JD, Robinson PC, Uldrick TS, Ljungman P (2021). COVID-19 in immunocompromised populations: implications for prognosis and repurposing of immunotherapies. J Immunother Cancer.

[3] Jakobsen NA, Vyas P (2018). From genomics to targeted treatment in haematological malignancies: a focus on acute myeloid leukaemia. Clin Med (Lond).

[4] Bedard PL, Hyman DM, Davids MS, Siu LL (2020). Small molecules, big impact: 20 years of targeted therapy in oncology. Lancet.

[5] Desai A, Yan Y, Gerson SL (2019). Concise reviews: cancer stem cell-targeted therapies: toward clinical success. Stem Cells Transl Med.

[6] Allegrezza MJ, Conejo-Garcia JR (2017). Targeted therapy and immunosuppression in the tumor microenvironment. Trends Cancer.

[7] Bechman K, Galloway JB, Winthrop KL (2019). Small-molecule protein kinases inhibitors and the risk of fungal infections. Curr Fungal Infect Rep.

[8] Teh BW, Tam CS, Handunnetti S, Worth LJ, Slavin MA (2018). Infections in patients with chronic lymphocytic leukaemia: mitigating risk in the era of targeted therapies. Blood Rev.

[9] Davis JS, Ferreira D, Paige E, Gedye C, Boyle M (2020). Infectious complications of biological and small molecule targeted immunomodulatory therapies. Clin Microbiol Rev.

[10] Tau N, Shargian-Alon L, Reich S, Paul M, Gafter-Gvili A, Shepshelovich D, Yahav D (2019). Reporting infections in clinical trials of patients with haematological malignancies. Clin Microbiol Infect.

[11] Cohen SB, Emery P, Greenwald MW (2006). Rituximab for rheumatoid arthritis refractory to anti-tumor necrosis factor therapy: results of a multicenter, randomized, double-blind, placebo-controlled, phase III trial evaluating primary efficacy and safety at twenty-four weeks. Arthritis Rheum.

[12] Aksoy S, Harputluoglu H, Kilickap S, Dede DS, Dizdar O, Altundag K, Barista I (2007). Rituximab-related viral infections in lymphoma patients. Leuk Lymphoma.

[13] Byrd JC, Brown JR, O'Brien S (2014). Ibrutinib versus ofatumumab in previously treated chronic lymphoid leukemia. N Engl J Med.

[14] Dimopoulos MA, Oriol A, Nahi H (2016). Daratumumab, lenalidomide, and dexamethasone for multiple myeloma. N Engl J Med.

[15] Palumbo A, Chanan-Khan A, Weisel K (2016). Daratumumab, bortezomib, and dexamethasone for multiple myeloma. N Engl J Med.

[16] Drgona L, Gudiol C, Lanini S, Salzberger B, Ippolito G, Mikulska M (2018). ESCMID Study Group for Infections in Compromised Hosts (ESGICH) Consensus Document on the safety of targeted and biological therapies: an infectious diseases perspective (Agents targeting lymphoid or myeloid cells surface antigens [II]: CD22, CD30, CD33, CD38, CD40, SLAMF-7 and CCR4). Clin Microbiol Infect.

[17] Stilgenbauer S, Zenz T, Winkler D (2009). Subcutaneous alemtuzumab in fludarabine-refractory chronic lymphocytic leukemia: clinical results and prognostic marker analyses from the CLL2H study of the German Chronic Lymphocytic Leukemia Study Group. J Clin Oncol.

[18] Agius MA, Klodowska-Duda G, Maciejowski M (2019). Safety and tolerability of inebilizumab (MEDI-551), an anti-CD19 monoclonal antibody, in patients with relapsing forms of multiple sclerosis: results from a phase 1 randomised, placebo-controlled, escalating intravenous and subcutaneous dose study. Mult Scler.

[19] Kantarjian HM, Stein AS, Bargou RC (2016). Blinatumomab treatment of older adults with relapsed/refractory B-precursor acute lymphoblastic leukemia: results from 2 phase 2 studies. Cancer.

[20] Trotman J, Opat S, Gottlieb D (2020). Zanubrutinib for the treatment of patients with Waldenström macroglobulinemia: 3 years of follow-up. Blood.

[21] Parmar S, Patel K, Pinilla-Ibarz J (2014). Ibrutinib (Imbruvica): a novel targeted therapy for chronic lymphocytic leukemia. P T.

[22] Marchesini G, Nadali G, Facchinelli D (2021). Infections in patients with lymphoproliferative diseases treated with targeted agents: SEIFEM multicentric retrospective study. Br J Haematol.

[23] Vannucchi AM, Kiladjian JJ, Griesshammer M (2015). Ruxolitinib versus standard therapy for the treatment of polycythemia vera. N Engl J Med.

[24] Davids MS, Hallek M, Wierda W (2018). Comprehensive safety analysis of venetoclax monotherapy for patients with relapsed/refractory chronic lymphocytic leukemia. Clin Cancer Res.

[25] DiNardo CD, Pratz KW, Letai A (2018). Safety and preliminary efficacy of venetoclax with decitabine or azacitidine in elderly patients with previously untreated acute myeloid leukaemia: a non-randomised, open-label, phase 1b study. Lancet Oncol.

[26] Lu RM, Hwang YC, Liu IJ, Lee CC, Tsai HZ, Li HJ, Wu HC (2020). Development of therapeutic antibodies for the treatment of diseases. J Biomed Sci.

[27] (2012). Monoclonal antibodies. LiverTox: Clinical and Research Information on Drug-Induced Liver Injury.

[28] Kaplon H, Chenoweth A, Crescioli S, Reichert JM (2022). Antibodies to watch in 2022. MAbs.

[29] Scheen AJ (2009). International classification of various types of monoclonal antibodies. (Article in French). Rev Med Liege.

[30] Harding FA, Stickler MM, Razo J, DuBridge RB (2010). The immunogenicity of humanized and fully human antibodies: residual immunogenicity resides in the CDR regions. MAbs.

[31] Mayrhofer P, Kunert R (2019). Nomenclature of humanized mAbs: early concepts, current challenges and future perspectives. Hum Antibodies.

[32] Payandeh Z, Bahrami AA, Hoseinpoor R (2019). The applications of anti-CD20 antibodies to treat various B cells disorders. Biomed Pharmacother.

[33] Sellebjerg F, Blinkenberg M, Sorensen PS (2020). Anti-CD20 monoclonal antibodies for relapsing and progressive multiple sclerosis. CNS Drugs.

[34] Marchesi F, Pimpinelli F, Giannarelli D (2022). Impact of anti-CD20 monoclonal antibodies on serologic response to BNT162b2 vaccine in B-cell Non-Hodgkin's lymphomas. Leukemia.

[35] Shah K, Cragg M, Leandro M, Reddy V (2021). Anti-CD20 monoclonal antibodies in systemic lupus erythematosus. Biologicals.

[36] Singh V, Gupta D, Almasan A (2015). Development of novel Anti-CD20 monoclonal antibodies and modulation in CD20 levels on cell surface: looking to improve immunotherapy response. J Cancer Sci Ther.

[37] Cooper N, Arnold DM (2010). The effect of rituximab on humoral and cell mediated immunity and infection in the treatment of autoimmune diseases. Br J Haematol.

[38] Madanchi N, Bitzan M, Takano T (2017). Rituximab in minimal change disease: mechanisms of action and hypotheses for future studies. Can J Kidney Health Dis.

[39] Lund FE, Randall TD (2010). Effector and regulatory B cells: modulators of CD4+ T cell immunity. Nat Rev Immunol.

[40] Casan JM, Wong J, Northcott MJ, Opat S (2018). Anti-CD20 monoclonal antibodies: reviewing a revolution. Hum Vaccin Immunother.

[41] Kater AP, Seymour JF, Hillmen P (2019). Fixed duration of Venetoclax-rituximab in relapsed/refractory chronic lymphocytic leukemia eradicates minimal residual disease and prolongs survival: post-treatment follow-up of the MURANO phase III study. J Clin Oncol.

[42] Luo C, Wu G, Huang X (2021). Efficacy and safety of new anti-CD20 monoclonal antibodies versus rituximab for induction therapy of CD20(+) B-cell non-Hodgkin lymphomas: a systematic review and meta-analysis. Sci Rep.

[43] Seymour JF, Kipps TJ, Eichhorst BF (2022). Enduring undetectable MRD and updated outcomes in relapsed/refractory CLL after fixed-duration venetoclax-rituximab. Blood.

[44] Brogan P, Yeung RS, Cleary G (2022). Phase IIa global study evaluating rituximab for the treatment of pediatric patients with granulomatosis with polyangiitis or microscopic polyangiitis. Arthritis Rheumatol.

[45] Tavakolpour S, Alesaeidi S, Darvishi M (2019). A comprehensive review of rituximab therapy in rheumatoid arthritis patients. Clin Rheumatol.

[46] Wise LM, Stohl W (2020). Belimumab and rituximab in systemic lupus erythematosus: a tale of two B cell-targeting agents. Front Med (Lausanne).

[47] Zonozi R, Wallace ZS, Laliberte K (2021). Incidence, clinical features, and outcomes of late-onset neutropenia from rituximab for autoimmune disease. Arthritis Rheumatol.

[48] Shimony S, Bar-Sever E, Berger T (2021). Late onset neutropenia after rituximab and obinutuzumab treatment - characteristics of a class-effect toxicity. Leuk Lymphoma.

[49] Smulski CR, Decossas M, Chekkat N (2017). Hetero-oligomerization between the TNF receptor superfamily members CD40, Fas and TRAILR2 modulate CD40 signalling. Cell Death Dis.

[50] Levit-Zerdoun E, Becker M, Pohlmeyer R (2016). Survival of Igα-deficient mature B cells requires BAFF-R function. J Immunol.

[51] Scapini P, Bazzoni F, Cassatella MA (2008). Regulation of B-cell-activating factor (BAFF)/B lymphocyte stimulator (BLyS) expression in human neutrophils. Immunol Lett.

[52] Tesfa D, Sander B, Lindkvist H (2021). The role of BAFF and G-CSF for rituximab-induced late-onset neutropenia (LON) in lymphomas. Med Oncol.

[53] Kridin K, Ahmed AR (2020). Post-rituximab immunoglobulin M (IgM) hypogammaglobulinemia. Autoimmun Rev.

[54] Tieu J, Smith RM, Gopaluni S (2021). Rituximab associated hypogammaglobulinemia in autoimmune disease. Front Immunol.

[55] Casulo C, Maragulia J, Zelenetz AD (2013). Incidence of hypogammaglobulinemia in patients receiving rituximab and the use of intravenous immunoglobulin for recurrent infections. Clin Lymphoma Myeloma Leuk.

[56] Arnold DM, Dentali F, Crowther MA (2007). Systematic review: efficacy and safety of rituximab for adults with idiopathic thrombocytopenic purpura. Ann Intern Med.

[57] Eichhorst B, Fink AM, Busch R (2014). Frontline Chemoimmunotherapy with Fludarabine (F), Cyclophosphamide (C), and rituximab (R) (FCR) shows superior efficacy in comparison to bendamustine (B) and rituximab (BR) in previously untreated and physically fit patients (pts) with advanced chronic lymphocytic leukemia (CLL): final analysis of an International, Randomized Study of the German CLL Study Group (GCLLSG) (CLL10 Study). Blood.

[58] Walker AR, Kleiner A, Rich L, Conners C, Fisher RI, Anolik J, Friedberg JW (2008). Profound hypogammaglobulinemia 7 years after treatment for indolent lymphoma. Cancer Invest.

[59] van Oers MH, Van Glabbeke M, Giurgea L (2010). Rituximab maintenance treatment of relapsed/resistant follicular non-Hodgkin's lymphoma: long-term outcome of the EORTC 20981 phase III randomized intergroup study. J Clin Oncol.

[60] Moulis G, Lapeyre-Mestre M, Palmaro A, Sailler L (2017). Infections in non-splenectomized persistent or chronic primary immune thrombocytopenia adults: risk factors and vaccination effect. J Thromb Haemost.

[61] Marignani M, Mangone M, Cox MC (2011). HCV-positive status and hepatitis flares in patients with B-cell non-Hodgkin's lymphoma treated with rituximab-containing regimens. Dig Liver Dis.

[62] Jiang X, Mei X, Feng D, Wang X (2015). Prophylaxis and treatment of Pneumocystis jiroveci pneumonia in lymphoma patients subjected to rituximab-contained therapy: a systemic review and meta-analysis. PLoS One.

[63] Barreto JN, Ice LL, Thompson CA (2016). Low incidence of pneumocystis pneumonia utilizing PCR-based diagnosis in patients with B-cell lymphoma receiving rituximab-containing combination chemotherapy. Am J Hematol.

[64] Maertens J, Cesaro S, Maschmeyer G (2016). ECIL guidelines for preventing Pneumocystis jirovecii pneumonia in patients with haematological malignancies and stem cell transplant recipients. J Antimicrob Chemother.

[65] Cheson BD, Leonard JP (2008). Monoclonal antibody therapy for B-cell non-Hodgkin's lymphoma. N Engl J Med.

[66] Goede V, Fischer K, Busch R (2014). Obinutuzumab plus chlorambucil in patients with CLL and coexisting conditions. N Engl J Med.

[67] Cinar OK, Marlais M, Al Obaidi M, Cheng IL, Tullus K, Brogan P, Moraitis E (2021). Ofatumumab use in juvenile systemic lupus erythematosus: a single centre experience. Lupus.

[68] Florou D, Katsara M, Feehan J, Dardiotis E, Apostolopoulos V (2020). Anti-CD20 agents for multiple sclerosis: spotlight on ocrelizumab and ofatumumab. Brain Sci.

[69] Davids MS, Kuss BJ, Hillmen P (2020). Efficacy and safety of duvelisib following disease progression on ofatumumab in patients with relapsed/refractory CLL or SLL in the DUO Crossover Extension Study. Clin Cancer Res.

[70] Desikan SP, Keating MJ, Ferrajoli A (2022). Early treatment with Ofatumumab in patients with high-risk CLL. Blood.

[71] Luan C, Chen B (2019). Clinical application of obinutuzumab for treating chronic lymphocytic leukemia. Drug Des Devel Ther.

[72] Al-Sawaf O, Zhang C, Tandon M (2020). Venetoclax plus obinutuzumab versus chlorambucil plus obinutuzumab for previously untreated chronic lymphocytic leukaemia (CLL14): follow-up results from a multicentre, open-label, randomised, phase 3 trial. Lancet Oncol.

[73] Kawasaki N, Yamashita-Kashima Y, Fujimura T, Yoshiura S, Harada N, Kondoh O, Yoshimura Y (2022). Resistance to obinutuzumab-induced antibody-dependent cellular cytotoxicity caused by abnormal Fas signaling is overcome by combination therapies. Mol Biol Rep.

[74] Marcus R, Davies A, Ando K (2017). Obinutuzumab for the first-line treatment of follicular lymphoma. N Engl J Med.

[75] Kotmayer L, László T, Mikala G (2023). Landscape of BCL2 resistance mutations in a real-world cohort of patients with relapsed/refractory chronic lymphocytic leukemia treated with Venetoclax. Int J Mol Sci.

[76] Jelínek T, Mihályová J, Hájek R (2018). CD38 targeted treatment for multiple myeloma. Vnitr Lek.

[77] Overdijk MB, Verploegen S, Bögels M (2015). Antibody-mediated phagocytosis contributes to the anti-tumor activity of the therapeutic antibody daratumumab in lymphoma and multiple myeloma. MAbs.

[78] Spencer A, Lentzsch S, Weisel K (2018). Daratumumab plus bortezomib and dexamethasone versus bortezomib and dexamethasone in relapsed or refractory multiple myeloma: updated analysis of CASTOR. Haematologica.

[79] Bahlis NJ, Dimopoulos MA, White DJ (2020). Daratumumab plus lenalidomide and dexamethasone in relapsed/refractory multiple myeloma: extended follow-up of POLLUX, a randomized, open-label, phase 3 study. Leukemia.

[80] Ruck T, Bittner S, Wiendl H, Meuth SG (2015). Alemtuzumab in multiple sclerosis: mechanism of action and beyond. Int J Mol Sci.

[81] Li Z, Richards S, Surks HK, Jacobs A, Panzara MA (2018). Clinical pharmacology of alemtuzumab, an anti-CD52 immunomodulator, in multiple sclerosis. Clin Exp Immunol.

[82] Rolla S, De Mercanti SF, Bardina V (2022). Long-term effects of alemtuzumab on CD4+ lymphocytes in multiple sclerosis patients: A 72-month follow-up. Front Immunol.

[83] Rasmussen TA, McMahon J, Chang JJ (2017). Impact of alemtuzumab on HIV persistence in an HIV-infected individual on antiretroviral therapy with Sezary syndrome. AIDS.

[84] Tsai YF, Hsu CM, Hsiao HH (2021). Management of hepatitis B virus reactivation in malignant lymphoma prior to immunosuppressive treatment. J Pers Med.

[85] Iannitto E, Minardi V, Calvaruso G (2005). Hepatitis B virus reactivation and alemtuzumab therapy. Eur J Haematol.

[86] Cohen JA, Coles AJ, Arnold DL (2012). Alemtuzumab versus interferon beta 1a as first-line treatment for patients with relapsing-remitting multiple sclerosis: a randomised controlled phase 3 trial. Lancet.

[87] Poh C, Shustov A, Huang IJ, Gopal AK, Smith SD (2022). Efficacy of short-duration alemtuzumab in T-cell large granular lymphocytic leukemia: potential for a response-adapted strategy. Blood.

[88] Roex MC, Wijnands C, Veld SA (2021). Effect of alemtuzumab-based T-cell depletion on graft compositional change in vitro and immune reconstitution early after allogeneic stem cell transplantation. Cytotherapy.

[89] O'Brien S, Ravandi F, Riehl T (2008). Valganciclovir prevents cytomegalovirus reactivation in patients receiving alemtuzumab-based therapy. Blood.

[90] Kim SJ, Moon JH, Kim H (2012). Non-bacterial infections in Asian patients treated with alemtuzumab: a retrospective study of the Asian Lymphoma Study Group. Leuk Lymphoma.

[91] Bosch W, Poowanawittayakom N, Chaikriangkrai K (2013). Tuberculous hepatitis in renal transplant recipients following alemtuzumab induction therapy. Transpl Infect Dis.

[92] Wray S, Havrdova E, Snydman DR (2019). Infection risk with alemtuzumab decreases over time: pooled analysis of 6-year data from the CAMMS223, CARE-MS I, and CARE-MS II studies and the CAMMS03409 extension study. Mult Scler.

[93] Sermer D, Elavalakanar P, Abramson JS, Palomba ML, Salles G, Arnason J (2023). Targeting CD19 for diffuse large B cell lymphoma in the era of CARs: other modes of transportation. Blood Rev.

[94] Topp MS, Kufer P, Gökbuget N (2011). Targeted therapy with the T-cell-engaging antibody blinatumomab of chemotherapy-refractory minimal residual disease in B-lineage acute lymphoblastic leukemia patients results in high response rate and prolonged leukemia-free survival. J Clin Oncol.

[95] Watkins MP, Bartlett NL (2018). CD19-targeted immunotherapies for treatment of patients with non-Hodgkin B-cell lymphomas. Expert Opin Investig Drugs.

[96] Zinzani PL, Minotti G (2022). Anti-CD19 monoclonal antibodies for the treatment of relapsed or refractory B-cell malignancies: a narrative review with focus on diffuse large B-cell lymphoma. J Cancer Res Clin Oncol.

[97] Mikulska M, Lanini S, Gudiol C, Drgona L, Ippolito G, Fernández-Ruiz M, Salzberger B (2018). ESCMID Study Group for Infections in Compromised Hosts (ESGICH) Consensus Document on the safety of targeted and biological therapies: an infectious diseases perspective (Agents targeting lymphoid cells surface antigens [I]: CD19, CD20 and CD52). Clin Microbiol Infect.

[98] Frampton JE (2020). Inebilizumab: first approval. Drugs.

[99] Davis JA, Shockley A, Glode AE (2022). Newly approved anti-CD19 monoclonal antibodies for the treatment of relapsed or refractory diffuse large B-cell lymphoma. J Oncol Pharm Pract.

[100] Wu J, Fu J, Zhang M, Liu D (2015). Blinatumomab: a bispecific T cell engager (BiTE) antibody against CD19/CD3 for refractory acute lymphoid leukemia. J Hematol Oncol.

[101] Golay J, D'Amico A, Borleri G (2014). A novel method using blinatumomab for efficient, clinical-grade expansion of polyclonal T cells for adoptive immunotherapy. J Immunol.

[102] Zugmaier G, Topp MS, Alekar S (2014). Long-term follow-up of serum immunoglobulin levels in blinatumomab-treated patients with minimal residual disease-positive B-precursor acute lymphoblastic leukemia. Blood Cancer J.

[103] Kantarjian H, Stein A, Gökbuget N (2017). Blinatumomab versus chemotherapy for advanced acute lymphoblastic leukemia. N Engl J Med.

[104] Apel A, Ofran Y, Wolach O (2020). Safety and efficacy of blinatumomab: A real world data. Ann Hematol.

[105] Shimada A (2019). Hematological malignancies and molecular targeting therapy. Eur J Pharmacol.

[106] Long M, Beckwith K, Do P (2017). Ibrutinib treatment improves T cell number and function in CLL patients. J Clin Invest.

[107] Parmar K, Thein K, Tijani L, Ball S (2022). PB1876: Acalabrutinib related infectious complications-a systematic review and meta-analysis of phase III RCT. HemaSphere.

[108] Tillman BF, Pauff JM, Satyanarayana G, Talbott M, Warner JL (2018). Systematic review of infectious events with the Bruton tyrosine kinase inhibitor ibrutinib in the treatment of hematologic malignancies. Eur J Haematol.

[109] Yang J, Nie J, Ma X, Wei Y, Peng Y, Wei X (2019). Targeting PI3K in cancer: Mechanisms and advances in clinical trials. Mol Cancer.

[110] Fruman DA, Chiu H, Hopkins BD, Bagrodia S, Cantley LC, Abraham RT (2017). The PI3K pathway in human disease. Cell.

[111] Benjamin DJ, Prasad V (2022). PI3K inhibitors in haematological malignancies. Lancet Oncol.

[112] Zirlik K, Veelken H (2018). Idelalisib. Recent Results Cancer Res.

[113] Cuneo A, Barosi G, Danesi R (2019). Management of adverse events associated with idelalisib treatment in chronic lymphocytic leukemia and follicular lymphoma: a multidisciplinary position paper. Hematol Oncol.

[114] Furman RR, Sharman JP, Coutre SE (2014). Idelalisib and rituximab in relapsed chronic lymphocytic leukemia. N Engl J Med.

[115] Zelenetz AD, Barrientos JC, Brown JR (2017). Idelalisib or placebo in combination with bendamustine and rituximab in patients with relapsed or refractory chronic lymphocytic leukaemia: interim results from a phase 3, randomised, double-blind, placebo-controlled trial. Lancet Oncol.

[116] Jones JA, Robak T, Brown JR (2017). Efficacy and safety of idelalisib in combination with ofatumumab for previously treated chronic lymphocytic leukaemia: an open-label, randomised phase 3 trial. Lancet Haematol.

[117] Brown JR, Furman RR, Flinn I (2013). Final results of a phase I study of idelalisib (GSE1101) a selective inhibitor of PI3Kδ, in patients with relapsed or refractory CLL. J Clin Oncol.

[118] Barrientos JC, Furman RF, Leonard J (2013). Update on a phase I study of the selective PI3Kδ inhibitor idelalisib (GS-1101) in combination with rituximab and/or bendamustine in patients with relapsed or refractory CLL. N Engl J Med.

[119] Tanase AD, Colita A, Craciun OG (2020). Allogeneic stem cell transplantation for adult T-cell leukemia/lymphoma-Romanian experience. J Clin Med.

[120] Bird ST, Tian F, Flowers N (2020). Idelalisib for treatment of relapsed follicular lymphoma and chronic lymphocytic leukemia: A comparison of treatment outcomes in clinical trial participants vs Medicare beneficiaries. JAMA Oncol.

[121] Cheah CY, Fowler NH (2016). Idelalisib in the management of lymphoma. Blood.

[122] Senkevitch E, Durum S (2017). The promise of Janus kinase inhibitors in the treatment of hematological malignancies. Cytokine.

[123] Ajayi S, Becker H, Reinhardt H, Engelhardt M, Zeiser R, von Bubnoff N, Wäsch R (2018). Ruxolitinib. Small Molecules in Hematology. Recent Results in Cancer Research.

[124] Verstovsek S, Mesa RA, Gotlib J (2017). Long-term treatment with ruxolitinib for patients with myelofibrosis: 5-year update from the randomized, double-blind, placebo-controlled, phase 3 COMFORT-I trial. J Hematol Oncol.

[125] Lussana F, Cattaneo M, Rambaldi A, Squizzato A (2018). Ruxolitinib-associated infections: a systematic review and meta-analysis. Am J Hematol.

[126] Kim YJ, Witwit H, Cubitt B, de la Torre JC (2021). Inhibitors of anti-apoptotic BCL-2 family proteins exhibit potent and broad-spectrum anti-mammarenavirus activity via cell cycle arrest at G0/G1 phase. J Virol.

[127] Mastalier B, Deaconescu V, Elaiah W (2015). Multiple Intestinal Lymphoma. Rom J Intern Med.

[128] Yi JH, Kim SJ, Kim WS (2017). Brentuximab vedotin: Clinical updates and practical guidance. Blood Res.

[129] Nademanee A, Sureda A, Stiff P (2018). Safety analysis of brentuximab vedotin from the phase III AETHERA trial in Hodgkin lymphoma in the post-transplant consolidation setting. Biol Blood Marrow Transplant.

[130] Pro B, Advani R, Brice P (2012). Brentuximab vedotin (SGN-35) in patients with relapsed or refractory systemic anaplastic large-cell lymphoma: results of a phase II study. J Clin Oncol.

[131] Tudesq JJ, Vincent L, Lebrun J (2017). Cytomegalovirus infection with retinitis after brentuximab vedotin treatment for CD30(+) lymphoma. Open Forum Infect Dis.

[132] Kantarjian HM, DeAngelo DJ, Stelljes M (2016). Inotuzumab ozogamicin versus standard therapy for acute lymphoblastic leukemia. N Engl J Med.

[133] Dahl J, Marx K, Jabbour E (2016). Inotuzumab ozogamicin in the treatment of acute lymphoblastic leukemia. Expert Rev Hematol.

[134] Kazi JU, Rönnstrand L (2019). FMS-like tyrosine kinase 3/FLT3: From basic science to clinical implications. Physiol Rev.

[135] Egbuna C, Patrick-Iwuanyanwu KC, Onyeike EN, Khan J, Alshehri B (2022). FMS-like tyrosine kinase-3 (FLT3) inhibitors with better binding affinity and ADMET properties than sorafenib and gilteritinib against acute myeloid leukemia: in silico studies. J Biomol Struct Dyn.

[136] Xu Q, He S, Yu L (2021). Clinical benefits and safety of FMS-like tyrosine kinase 3 inhibitors in various treatment stages of acute myeloid leukemia: a systematic review, meta-analysis, and network meta-analysis. Front Oncol.

[137] Buclin T, Thoma Y, Widmer N, André P, Guidi M, Csajka C, Decosterd LA (2020). The steps to therapeutic drug monitoring: a structured approach illustrated with imatinib. Front Pharmacol.

[138] Kalmanti L, Saussele S, Lauseker M (2015). Safety and efficacy of imatinib in CML over a period of 10 years: data from the randomized CML-study IV. Leukemia.

[139] McMurry H, Fletcher L, Traer E (2021). IDH inhibitors in AML-promise and pitfalls. Curr Hematol Malig Rep.

[140] Stein EM, Fathi AT, DiNardo CD (2020). Enasidenib in patients with mutant IDH2 myelodysplastic syndromes: a phase 1 subgroup analysis of the multicentre, AG221-C-001 trial. Lancet Haematol.

[141] Mato AR, Roeker LE, Lamanna N (2020). Outcomes of COVID-19 in patients with CLL: a multicenter international experience. Blood.

[142] Scarfò L, Chatzikonstantinou T, Rigolin GM (2020). COVID-19 severity and mortality in patients with chronic lymphocytic leukemia: a joint study by ERIC, the European Research Initiative on CLL, and CLL Campus. Leukemia.

[143] England JT, Abdulla A, Biggs CM (2021). Weathering the COVID-19 storm: lessons from hematologic cytokine syndromes. Blood Rev.

[144] Coutre SE, Barnett C, Osiyemi O (2022). Ibrutinib for hospitalized adults with severe coronavirus disease 2019 infection: results of the randomized, double-blind, placebo-controlled iNSPIRE study. Open Forum Infect Dis.

[145] Shen Y, Freeman JA, Holland J (2022). COVID‐19 vaccine failure in chronic lymphocytic leukaemia and monoclonal B‐lymphocytosis; humoural and cellular immunity. Br J Haematol.

[146] Parry H, McIlroy G, Bruton R (2021). Antibody responses after first and second Covid-19 vaccination in patients with chronic lymphocytic leukaemia. Blood Cancer J.

[147] Herishanu Y, Rahav G, Levi S (2022). Efficacy of a third BNT162b2 mRNA COVID-19 vaccine dose in patients with CLL who failed standard 2-dose vaccination. Blood.

[148] Blixt L, Bogdanovic G, Buggert M (2022). Covid-19 in patients with chronic lymphocytic leukemia: clinical outcome and B- and T-cell immunity during 13 months in consecutive patients. Leukemia.

